# Phosphoproteomic analysis reveals the diversity of signaling behind ErbB‐inhibitor‐induced phenotypes

**DOI:** 10.1111/febs.70197

**Published:** 2025-07-24

**Authors:** Katri Vaparanta, Zejia Song, Iman Farahani, Anne Jokilammi, Johannes Merilahti, Johanna Örling, Noora Virtanen, Pekka Haapaniemi, Cecilia Sahlgren, Klaus Elenius, Ilkka Paatero

**Affiliations:** ^1^ Medicity Research Laboratories and Institute of Biomedicine University of Turku Finland; ^2^ Turku Bioscience Centre University of Turku and Åbo Akademi University Turku Finland; ^3^ InFLAMES Research Flagship University of Turku and Åbo Akademi University Turku Finland; ^4^ Turku Doctoral Programme of Molecular Medicine (TuDMM) University of Turku Finland; ^5^ Biosciences, Faculty of Science and Engineering Åbo Akademi University Turku Finland; ^6^ Department of Oncology Turku University Hospital Finland

**Keywords:** ErbB inhibitor, phosphoproteome, signaling pathways, zebrafish embryos

## Abstract

The impact of kinase inhibitors on the phosphoproteome has been rarely investigated at a whole‐organism level. Here, we performed a phosphoproteomic analysis in embryonic zebrafish to identify the signaling pathways perturbed by ErbB receptor tyrosine‐protein kinase inhibitors gefitinib, lapatinib, and AG1478 at the organism level. The phosphorylation of proteins associated with the phosphoinositide 3‐kinase (PI3K)/protein kinase B (Akt), p38 mitogen‐activated protein kinase (MAPK), Notch, Hippo/Yap, and β‐catenin signaling pathways was differentially regulated by the ErbB inhibitors. Gene set enrichment analyses indicated differential neurological and myocardial phenotypes of different ErbB inhibitors. To assess the neurological and myocardial effects, motility and ventricle growth assays were performed with inhibitor‐treated embryos. The treatment with the inhibitors targeting the PI3K/Akt, p38 MAPK, and Notch signaling pathways, along with the ErbB inhibitors AG1478 and lapatinib, perturbed the overall movement and ventricle wall growth of zebrafish embryos. Taken together, these results indicate that inhibitors with overlapping primary targets can affect different signaling pathways while eliciting similar physiological phenotypes.

## Graphical abstract

Whole‐organism phosphoproteomics after ErbB inhibitor treatment was assessed in zebrafish embryos. Differential signaling was uncovered after treatments with more selective ErbB inhibitors and pan‐ErbB inhibitors, despite similar neurological and myocardial phenotypes. Inhibition of the differentially regulated pathways produced similar phenotypes to the ErbB inhibitor treatments. The results suggest that ErbB inhibitors may induce similar phenotypes by perturbing diverse signaling pathways.

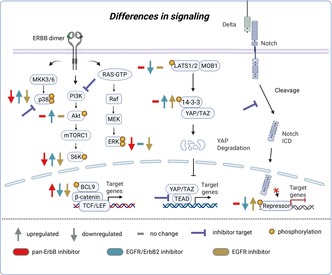

**Abbreviations**
Akt: protein kinase B
Dpf: days post fertilization
EGFR: epidermal growth factor receptor
ERK: extracellular signal regulated kinase
GPF: gas phase fractionation
GSEA: gene set enrichment analysis
JAK: janus kinase signal transducer and activator of transcription
KEGG: kyoto encyclopedia of genes and genomes
LC‐ESI‐MS/MS: liquid chromatography electrospray ionization tandem mass spectrometry
MAPK: mitogen‐activated protein kinase
MEK: mitogen‐activated protein kinase kinase
MS/MS: tandem mass spectrometry
mTORC1: mechanistic target of rapamycin complex 1
PI3K: phosphoinositide 3 kinase
PLCγ: phospholipase C gamma
YAP: yes‐associated protein

## Introduction

Cancer treatment has seen the development of molecularly targeted therapies, which act by perturbing cellular signaling systems and pathways [1]. The ErbB/Her receptor tyrosine kinases were among the first signaling molecules to be targeted for cancer therapy [1]. Currently, several antibodies and small molecules targeting ErbB signaling have been developed with differential effects in clinical trials [2, 3], indicating differences in the mechanism of action and/or toxicity profiles. Compounds that selectively target either epidermal growth factor receptor (EGFR), EGFR, and ErbB2 or all ErbB receptors with a functional kinase domain (EGFR, ErbB2, and ErbB4) have been developed [2, 3]. The ErbB receptor tyrosine kinases are activated by ligand binding and dimerization, and mainly mediate their effects through the activation of phosphatidylinositol 3‐kinase (PI3K)/protein kinase B (Akt) and mitogen‐activated protein kinase kinase (MEK)/extracellular signal‐regulated kinase (Erk) signaling [4, 5, 6]. Several other signaling pathways including other mitogen‐activated protein kinase (MAPK), the Janus kinase/signal transducer and activator of transcription (JAK/STAT), phospholipase C gamma (PLCγ), and VAV/Rho/Rac signaling pathways are known to be regulated by the ErbB kinases [4, 5, 6]. Embryonic knockouts of the ErbB genes are lethal and have revealed the pivotal role of these receptors in the development of the neural, cardiac, neuromuscular, and skeletal system, and the epithelial layers of the kidneys, lungs, gastrointestinal tract, eyes, and skin [7, 8, 9, 10, 11, 12, 13, 14]. Variation between the knockout phenotypes has been observed depending on the targeted receptor gene and the mice strain. The cardiac phenotype has been observed only when either the ErbB2 or ErbB4 gene has been knocked out, and the epithelial layers mostly affected by EGFR deletion have shown significant variability between mice strains. Oncogenic mutations, amplifications, and gene variations of the ErbB receptors are frequent in glioblastoma, lung, breast, ovarian, stomach, head and neck, and colorectal cancers [3].

Interestingly, compounds with similar primary targets and effects *in vitro* may have different effects *in vivo*. This indicates that *in vivo* analysis of the effects of compounds on cell signaling could be used to elucidate the organismal level changes in their actions. This is, however, difficult to achieve using mammalian *in vivo* models or human samples, as already the size and complexity hinder effective whole‐organism analyses of signaling effects. In recent years, zebrafish embryos have been utilized increasingly as an *in vivo* model to analyze the *in vivo* effects of compounds [15]. The small size and the ability to breed a large number of zebrafish embryos in a short period enable analysis of signaling effects at the whole‐organism level [16, 17] without the need for prior selection of tissues or cell types of interest. This could provide a more comprehensive view of the effects on the organismal level, opening new insight into the biological actions of compounds. This approach can identify the pathways most broadly impacted by a chemical compound, which may differ from those responsible for the drug's therapeutic effect in the target tissue. By identifying the key perturbed pathways, it may be easier to recognize the organs mainly affected by the compound treatment as well as the mechanisms for pharmacological effect and organ toxicity. Mass‐spectrometric analysis of the phosphoproteome has been, however, underutilized in studies of the effects of chemical compounds on zebrafish development and signaling.

Phenotypic analyses of zebrafish embryos exposed to ErbB inhibitors lapatinib, gefitinib, or AG1478 have shown differential phenotypes in cardiac biology and in the overall movement of the zebrafish embryos [18, 19]. Both the ventricle wall growth and the overall movement of the zebrafish embryos have been strongly affected by AG1478 and lapatinib treatment and uninfluenced by gefitinib treatment [18, 19]. Gefitinib is more selective to EGFR, whereas lapatinib (EGFR/ERBB2 inhibitor) and AG1478 (pan‐ERBB inhibitor) inhibit ErbB receptors more broadly (Table 1). To investigate the signaling differences underlying observed phenotypic variations and how inhibitor target selectivity affects organism‐wide signaling, we performed an in‐depth whole‐organism phosphoproteomic analysis with protein mass spectrometry.

**Table 1:** Selectivity of the ERBB kinase inhibitors used in this study. EC100, maximal effective concentration; IC50, half maximal inhibitory concentration; *K*_d_, binding constant; NA, information not available.

Inhibitor	Metric	EGFR	ERBB2	ERBB3	ERBB4	Non‐ERBB targets^a^	Reference
Erlotinib	*K*_d_ (nm)	0.67	2900	1100	230	2055 [3.1, 9600]	[70]
Gefitinib	*K*_d_ (nm)	0.57	3500	790	410	2402 [13, 7500]	[70]
Afatinib	*K*_d_ (nm)	0.25	5	4500	6.3	1863 [79, 6200]	[70]
Neratinib	*K*_d_ (nm)	1.1	6	7.7	2.4	2405 [0.65, 8400]	[70]
Lapatinib	*K*_d_ (nm)	2.4	7	5500	54	2974 [670, 7500]	[70]
AG‐825	IC50 (μm)	19	0.15	NA	NA	72.5 [40, 100]	[71]
AG‐1478	EC100 (μm)	0.5	0.66	NA	1	NA	[72]

^a^ Mean [minimum, maximum] values are shown.

## Results

**Fig. 1:**
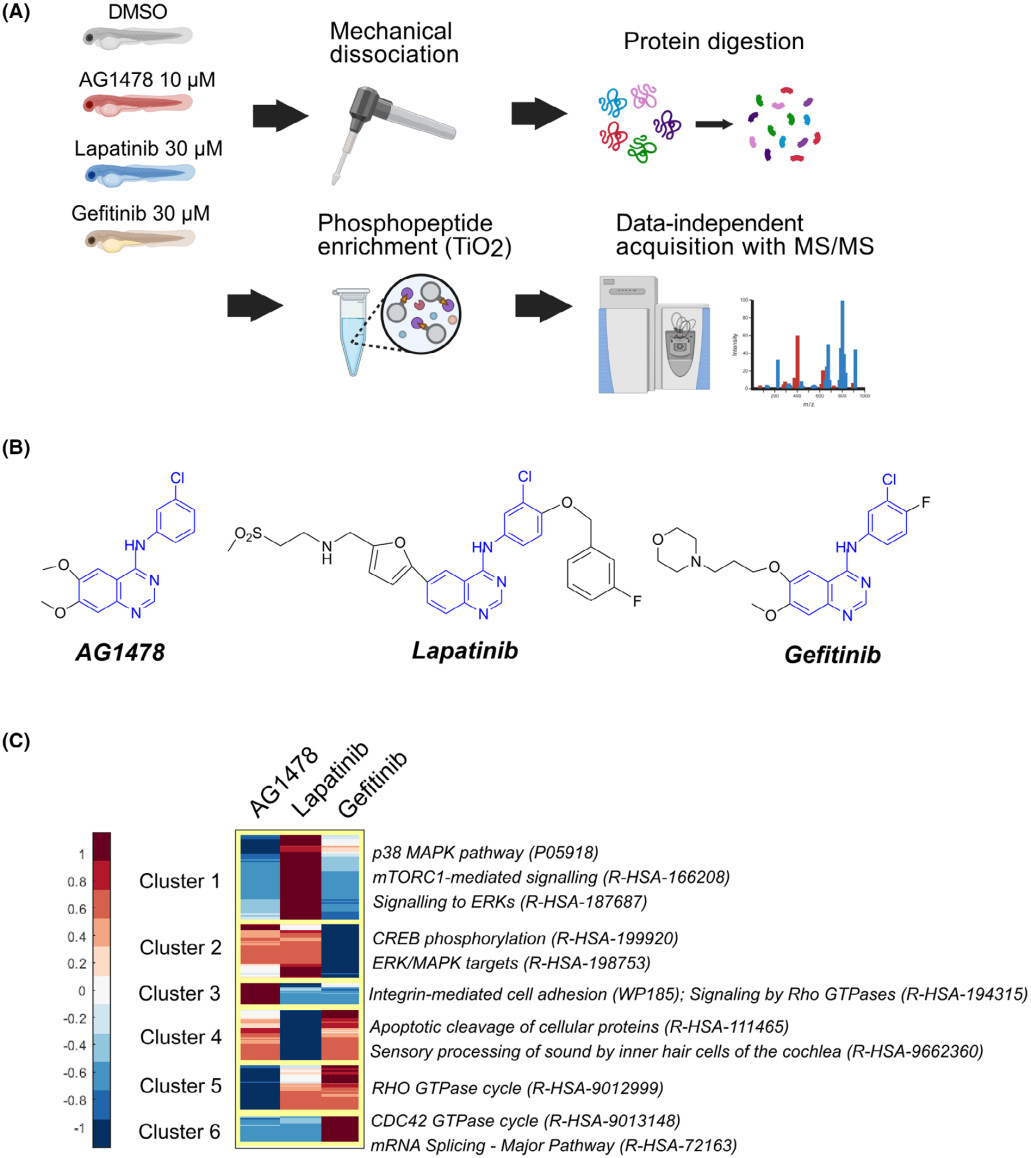
Whole‐organism phosphoproteome of ErbB inhibitor‐treated zebrafish embryos. (A) The workflow for the global phosphoproteome acquirement from ErbB inhibitor‐treated zebrafish embryos. (B) The chemical structure of the ErbB inhibitors. The shared core structure is indicated in blue. (C) Heatmap of the differently regulated phosphopeptides. The median fold change against the control DMSO treatment of four replicate experiments is visualized on a standardized scale. The clusters were identified with hierarchical clustering. Overrepresentation testing was used to associate pathways for each cluster.

To explore the global phosphoproteomic changes induced by the ErbB kinase inhibitors reported to have differential target selectivity, off‐target profiles (Table 1), and phenotypic effects, we exposed developing zebrafish embryos to lapatinib, gefitinib, AG1478, or DMSO for 1 h (Fig. 1A). Inhibitor concentrations previously noted to have a phenotypic effect but no significant mortality in the zebrafish embryos were used [18, 19]. To reduce the risk of malformations and subsequent unspecific secondary effects on cell signaling, the inhibitor exposures were started at 3 dpf (for signaling assays) or 2 dpf (for phenotypic assays) when the major morphogenetic events, such as gastrulation, segmentation, and initiation of organogenesis, had already been completed [20]. The phosphopeptides from the embryos were enriched and analyzed with protein mass spectrometry using data‐independent acquisition with tandem mass spectrometry (MS/MS) [21, 22]. In total, 23 141 unique phosphopeptides were detected (Table S1), and the quantities of 2033 phosphopeptides were significantly altered between treatments (Table S2). Differential expression analysis on the phosphorylated peptides in each condition was conducted, and hierarchical clustering was utilized to identify differentially regulated clusters of phosphopeptides. Even though the inhibitors are structurally related (Fig. 1B), apparent differences in the size of the phosphopeptide clusters were observed (Fig. 1C). A large cluster of phosphopeptides that were enriched in lapatinib‐treated embryos but reduced in AG1478 and gefitinib‐treated embryos was detected (Cluster 1 in Fig. 1C). Overrepresentation analysis indicated that phosphopeptides in this cluster were associated with the p38 MAPK, ERK, and mechanistic target of rapamycin complex 1 (mTORC1) pathways (Fig. 1C). The clusters of phosphopeptides that were enriched only in either AG1478 (Cluster 3 in Fig. 1C) or gefitinib (Cluster 6 in Fig. 1C) treated embryos but reduced in embryos treated with the other inhibitors were, in turn, significantly smaller.

**Fig. 2:**
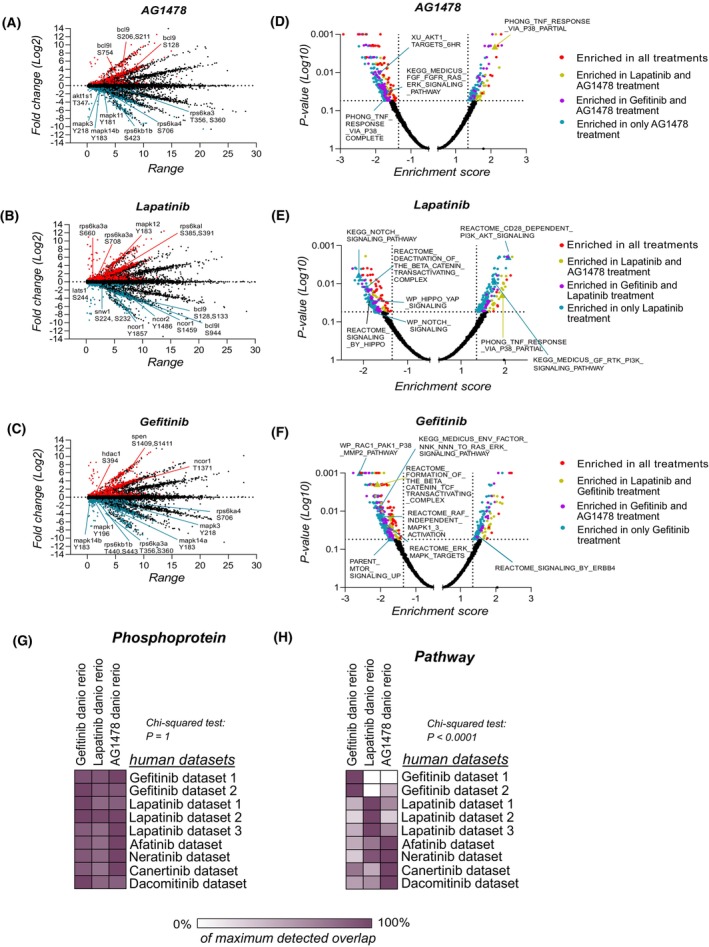
Phosphorylation of proteins involved in the Notch, Erk MAPK, PI3K/Akt/mTOR, p38 MAPK, and β‐catenin signaling pathways is differently regulated by the ErbB inhibitors. (A–C) Scatter plots of the identified phosphopeptides from the MS/MS analysis in each condition. The significantly downregulated (blue) and upregulated (red) phosphopeptides are indicated. Median fold change against the DMSO control treatment and the value range of four replicate experiments in log_2_ scale are visualized in the *y*‐ and *x*‐axis, respectively. (D–F) Volcano plots of the results of the gene set enrichment analysis performed on the global phosphoproteomics data. (G) Overlap of the differentially phosphorylated proteins between the MS/MS analysis results and previously acquired human datasets in Refs [62, 63, 64, 65, 66, 67] and ProteomeXchange Consortium (http://proteomecentral.proteomexchange.org; datasets PXD004007, PXD000375, PXD018046, PXD017985, PXD005336, PXD004373) of the ErbB inhibitor‐regulated phosphoproteome. (H) Overlap of the overrepresented pathways of the differentially phosphorylated proteins between the MS/MS analysis results and previously acquired human datasets [62, 63, 64, 65, 66, 67] of ErbB inhibitor‐regulated phosphoproteome.

The differentially regulated individual phosphorylated peptides in each condition were visualized in scatter plots (Fig. 2A–C). In AG1478 and gefitinib‐treated embryos, the downregulation of the phosphorylation of the activating residues of ERK MAPKs (Mapk1/3) and p38 MAPKs (Mapk11/12/13/14) was observed along with the phosphorylation of S6 kinases (Rps6ks) and other substrates (Akt1s1) downstream of the Akt/mTOR pathway. In the lapatinib‐treated embryos, the phosphorylation of both p38 MAPKs and S6 kinases was upregulated. In the lapatinib‐treated embryos, the phosphorylation of proteins involved in the Hippo/Yes‐associated protein (YAP) pathway (Lats1), transcriptional repressors of Notch signaling (Ncor1/2, Snw1), and transcriptional activators of β‐catenin (Bcl9/Bcl9l) was downregulated. In gefitinib‐treated embryos, however, the phosphorylation of the transcriptional repressors of Notch signaling (Hdac1, Spen, Ncor1) was upregulated. In AG1478‐treated embryos, the phosphorylation of the transcriptional activators of β‐catenin (Bcl9/Bcl9l) was significantly increased. Gene set enrichment analyses (GSEAs) [23] were performed to identify the dysregulated signaling pathways from the whole dataset (Fig. 2D–F, Table S3). The phosphorylation events associated with the p38 MAPK and PI3K/Akt/mTOR signaling pathways were discovered to be upregulated by lapatinib and downregulated by AG1478 and gefitinib treatment (Fig. 2D–F). The phosphorylation of proteins involved in ERK MAPK signaling was downregulated by gefitinib and AG1478 treatment (Fig. 2D,F) while the phosphorylation of proteins involved in Hippo/YAP and Notch signaling was downregulated by lapatinib treatment only (Fig. 2E). Transcriptional β‐catenin signaling was downregulated by gefitinib and lapatinib, but not by AG1478. Taken together, these results suggest that multiple signaling pathways are differently regulated by gefitinib, lapatinib, and AG1478 treatment.

Since these compounds are designed to inhibit the human ErbB kinases, the targets of these compounds could have differences between zebrafish and humans. To test this, a multiple sequence alignment on the kinase domain of ErbB receptors that binds these compounds was performed. The ErbB kinase domains had high amino acid sequence identity (86 ± 9%, Table S4) between human and zebrafish ErbB proteins, indicating conservation of the drug targets. To further compare the effects in zebrafish vs. human, the overlap of the results of the phosphoproteomic analysis in zebrafish with published datasets from ErbB inhibitor‐treated human samples was explored both at the phosphoprotein and signaling pathway level. Analysis on the phosphoprotein level indicated only limited specificity of overlap between different ErbB inhibitors (Fig. 2G, Table S6). However, pronounced differences in the overlap of signaling pathways were discovered between ErbB inhibitor treatments (Fig. 2H, Table S6). Significantly more overlap between the signaling pathways of zebrafish samples treated with gefitinib and the human samples treated with gefitinib than with the human samples treated with lapatinib or with the pan‐ErbB inhibitors (afatinib, neratinib, canertinib, and dacomitinib) was observed. Similarly, the signaling pathways perturbed in zebrafish embryos by lapatinib treatment or AG1478 treatment overlapped most with the signaling pathways perturbed in the human samples treated with lapatinib or pan‐ErbB inhibitors, respectively. This indicates that the signaling differences observed in the human samples treated with these inhibitors are recapitulated in the zebrafish context on the signaling pathway level.

**Fig. 3:**
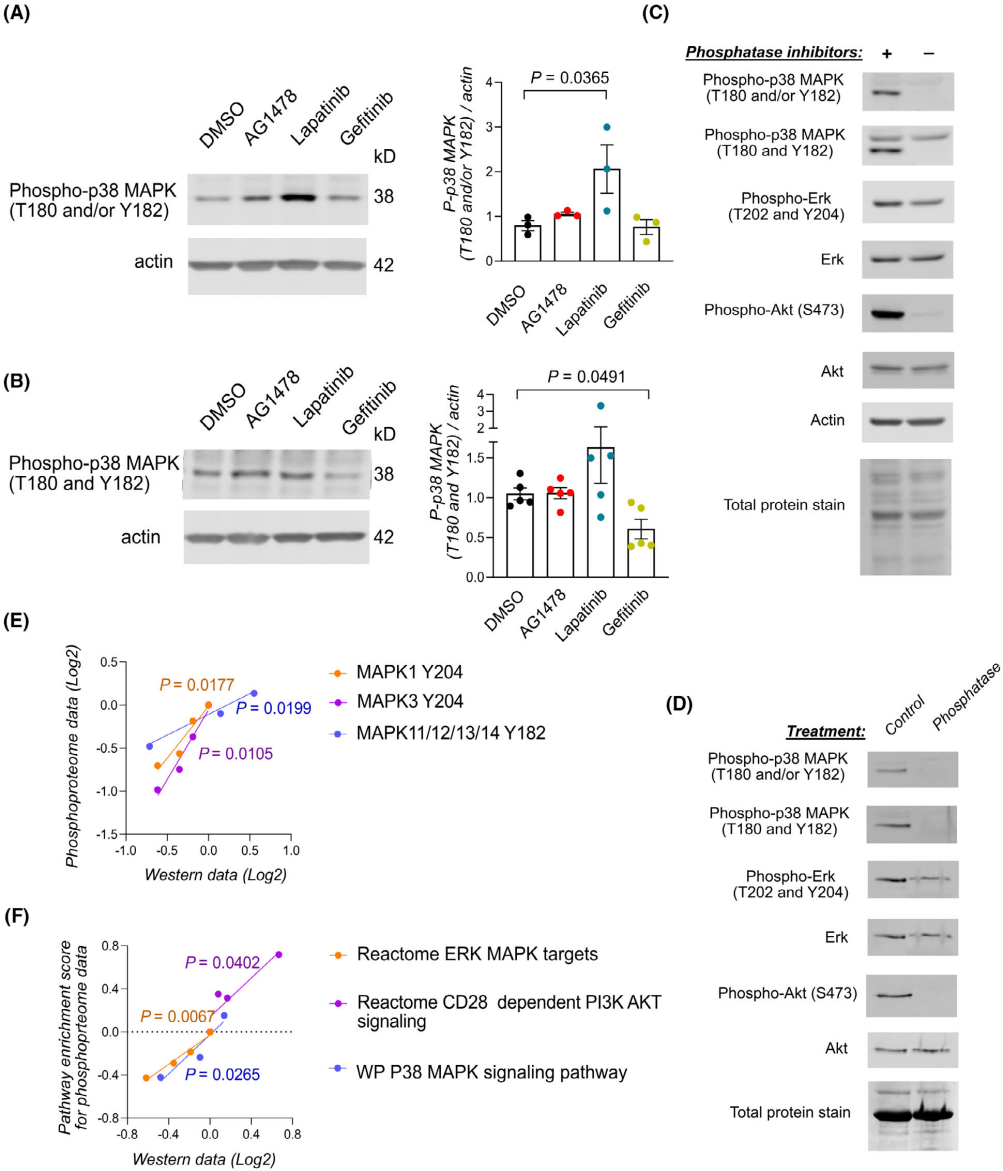
Validation of the ErbB inhibitor‐induced signaling changes identified through the whole‐organism phosphoproteomic analysis. (A, B) Representative blots and densitometric quantification of the western analyses on the activation of p38 MAPK kinases in ErbB inhibitor‐treated zebrafish embryos at 3 dpf. Mean ± SEM. One dot represents one replicate experiment (3 experiments in A and 5 experiments in B) One‐way ANOVA (A) and Welch's ANOVA (B) were used for statistical testing. Dunnett's multicomparison test was utilized for ANOVA *post hoc* analyses. (C) Untreated zebrafish embryos of 4 dpf were lysed with an electronic pestle into a lysis buffer with and without phosphatase inhibitors to extract proteins. The protein lysates were incubated at +37 °C for 90 min to activate endogenous phosphatases and subjected to western analysis. The membranes were probed with the indicated antibodies. The experiment was repeated twice. (D) Untreated zebrafish embryos of 4 dpf were lysed with an electronic pestle into a lysis buffer without phosphatase inhibitors to extract proteins. Two reaction mixtures of equal protein concentration were made, one where all reactants of the FastAP reaction were included and one where the alkaline phosphatase was omitted. The reaction mixture with the alkaline phosphatase was incubated at +37 °C for 90 min. Both reaction mixtures were subjected to western analysis, and the membranes were probed with the indicated antibodies. The experiment was done once. (E, F) Correlation (*n* = 4) of previously acquired [18] and new western analyses with the global phosphoproteome data at the phosphosite level (E) and with the GSEA enrichment scores of the pathways enriched in the phosphoproteome data (F). The mean LFQ‐intensity values of the phosphopeptides containing the phosphorylated residue and densitometric measurements of the western analyses were normalized against the DMSO control and transformed to the Log_2_ scale. One‐tailed Pearson's correlation analysis was utilized. One dot represents the value of one treatment condition (DMSO, AG1478, lapatinib, gefitinib) in the western and MS/MS or gene set enrichment analyses. Only *P*‐values of *P* < 0.10 are plotted in the graphs.

To validate the findings of the phosphoproteomic analysis, the phosphorylation of the two activating residues of p38 MAPKs was measured in gefitinib, lapatinib, AG1478, and control‐treated zebrafish embryos with western analyses. As expected from the results of the phosphoproteomic analysis, the overall (Fig. 3A, Table S6) or simultaneous double (Fig. 3B, Table S6) phosphorylation of the activating residues of p38 MAPKs was increased by lapatinib treatment and decreased by gefitinib treatment compared to other treatments (Fig. 3A,B). To further confirm this data, we carried out experiments with zebrafish embryo lysates treated with or without phosphatase inhibitors. The signal intensity of anti‐phospho‐p38 and anti‐phospho‐Akt antibodies strongly reduced, and the intensity of the anti‐phospho‐Erk signal halved when phosphatase inhibitors were omitted (Fig. 3C). This indicates that these antibodies indeed detected phosphorylated proteins in zebrafish lysates. Similar results were obtained when embryo lysates were treated with a recombinant phosphatase (Fig. 3D). To examine the correlation of previously [18] and newly executed western analyses with the results of the phosphoproteomic analysis, correlation analyses were performed. The intensity values of phosphosite‐specific antibodies in western analyses had a significant positive correlation with the intensity values of the phosphopeptides identified in the phosphoproteomic analysis (Fig. 3E) and the enrichment scores of signaling pathways from the GSEAs that were performed on the phosphoproteomics data (Fig. 3F). This suggests that the phosphoproteomic analysis reliably measured signaling activities in zebrafish embryos.

**Fig. 4:**
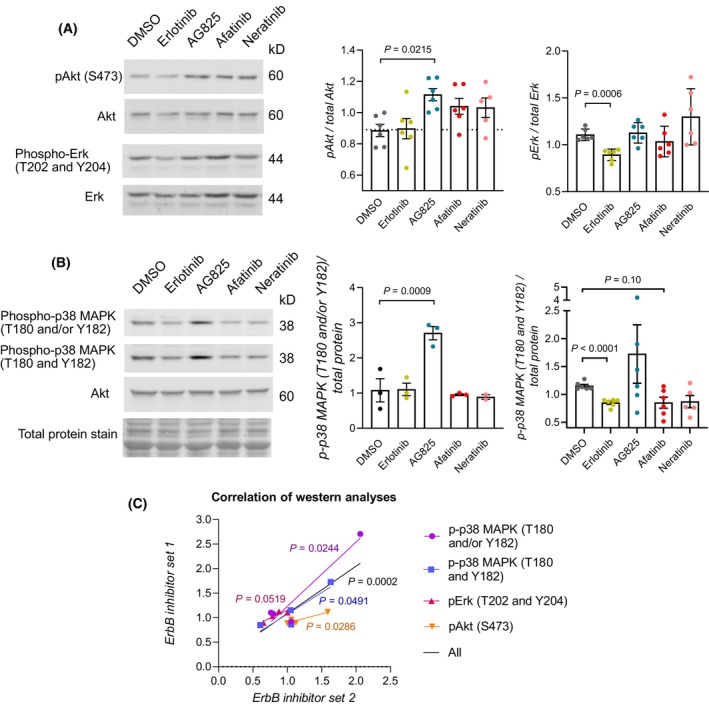
Phosphoproteomic changes in zebrafish embryos treated with another set of ERBB inhibitors. Zebrafish embryos at 3 dpf were treated with 20 μm of Erlotinib (EGFR inhibitor), AG825 (EGFR/ERBB2 inhibitor), Afatinib (pan‐ERBB inhibitor), Neratinib (pan‐ERBB inhibitor) or 1% DMSO for 1 h and dissociated. The lysed embryos were subjected to Western analysis. (A) Representative blots and densitometric quantification of the western analyses on the activation of Akt and Erk in ErbB inhibitor‐treated zebrafish embryos. Mean ± SEM (Akt) and Mean ± SD (Erk). One dot represents one replicate experiment (*n* = 6). One‐way ANOVA (Akt) and Welch's ANOVA (Erk) were used for statistical testing. Dunnett's multicomparison test was utilized for ANOVA *post hoc* analyses. (B) Representative blots and densitometric quantification of the western analyses on the activation of p38 MAPKs in ErbB inhibitor‐treated zebrafish embryos. Mean ± SEM. One dot represents one replicate experiment (*n* = 3 (middle panel) or *n* = 6 (right panel)). One‐way ANOVA (T180 and/or Y182) and Brow‐Forsythe and Welch's ANOVA (T180 and Y182) were used for statistical testing. Dunnett's and Dunn's multicomparison tests were utilized for ANOVA *post hoc* analyses, respectively. (C) Correlation (*n* = 4) of the proteomics changes detected with western analyses from zebrafish embryos treated with the first set (Gefitinib, Lapatinib, AG1478) and second set (Erlotinib, AG825, Afatinib and Neratinib) of ERBB inhibitors. The results from inhibitor treatments were matched based on reported ERBB inhibitor selectivity (Table 1; DMSO with DMSO, Gefitinib with Erlotinib, Lapatinib with AG825, AG1478 with Afatinib and Neratinib). One‐tailed Pearson correlation analysis. Only *P*‐values of *P* < 0.10 are plotted in the graphs.

As an additional control, we treated zebrafish embryos with ErbB inhibitors with similar ErbB selectivity (Table 1). We used erlotinib as a control for gefitinib, AG825 for lapatinib, and afatinib and neratinib for AG1478. The phosphoproteomic changes induced by inhibitors with similar ErbB family kinase selectivity significantly correlated (*P* = 0.0002, *r*: 0.80, *R* ^2^: 0.65, Fig. 4A–C, Table S6) indicating that the ErbB selectivity plays a major role in determining the phosphoproteomic changes induced by these inhibitors.

**Fig. 5:**
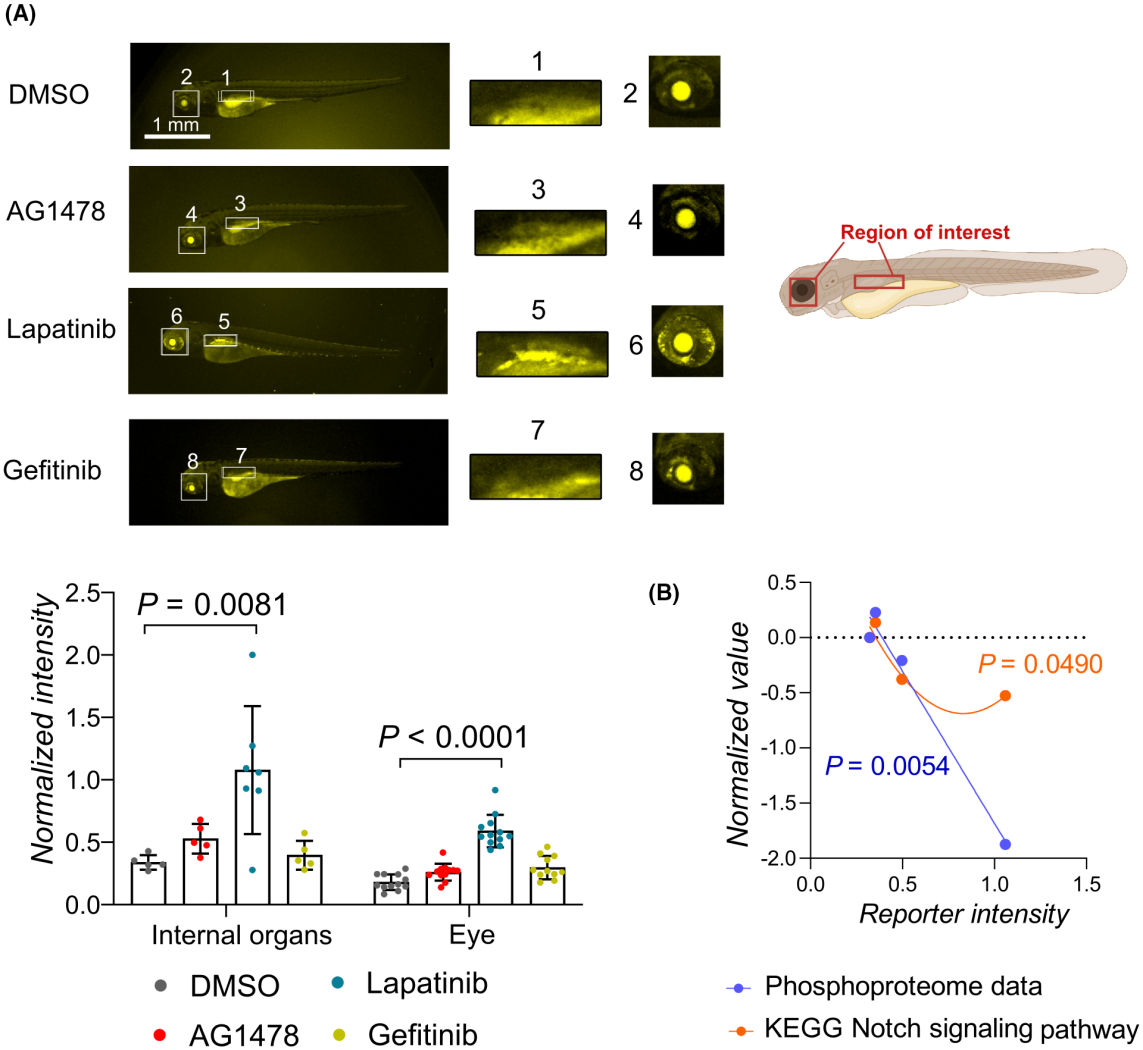
Notch signaling in zebrafish embryos treated with ERBB inhibitors. (A) Fluorescence microscope images and intensity measurements of the tp1:Venus‐pest Notch reporter line [24] zebrafish embryos treated with the indicated ErbB inhibitors or DMSO at 3 dpf. The regions of interest have been outlined in the schematic. Kruskal–Wallis ANOVA and Dunn's multicomparison test were utilized for statistical analysis. One dot corresponds to one zebrafish embryo (Internal organs: DMSO, *n* = 5; AG1478, *n* = 5; lapatinib, *n* = 7; gefitinib, *n* = 5. Eye: DMSO, *n* = 11, AG1478, *n* = 12; lapatinib, *n* = 12; gefitinib, *n* = 11). Mean ± SD. Scale bar, 1 mm. (B) Correlation and nonlinear relationship analysis of the mean LFQ intensity of the phosphopeptides of proteins involved in the Notch signaling pathway or the GSEA enrichment scores and the Notch reporter fluorescence intensity values. One‐tailed Pearson correlation and least squares fitting (*n* = 4) were used for the analyses. The statistical significance of the *R* ^2^ for the quadratic fit was calculated with an *F*‐test. One dot represents the value of one treatment condition (DMSO, AG1478, lapatinib, gefitinib) in the Notch reporter and MS/MS or gene set enrichment analyses. Only *P*‐values of *P* < 0.10 are plotted in the graphs.

Since the differential effect on Notch signaling by the ErbB inhibitors was suggested by the GSEA, the effect of ErbB inhibitor treatment on the transcriptional activity of Notch was measured as an additional control by utilizing a transgenic Notch activity reporter zebrafish line (*tp1:VENUS‐PEST*) [24]. The signal from the reporter has been detected in several tissues in the zebrafish embryos, including the eye [25] and several internal organs such as the pancreas at 2–4 dpf [26] and in the liver at 4 dpf [27]. The fluorescence signal from the Notch reporter was observed to be more intense in the eye and internal organs in the lapatinib‐treated embryos compared to gefitinib, AG1478, or control‐treated embryos (Fig. 5A, Table S6). This suggests that the reduced phosphorylation of the transcriptional repressors of Notch signaling by lapatinib treatment increased the transcriptional activity of Notch in the embryos. Overall, the embryos treated with ErbB inhibitors from 2 to 4 dpf showed normal gross morphology. As the measurements of reporter fluorescence could have been affected by the autofluorescence of yolk and iridophores, we aimed to confirm the regulation of the Notch pathway by another method. The relationship of the observed fluorescent signal with the intensity values of the phosphopeptides of transcriptional Notch repressors identified in the phosphoproteomic analysis and the enrichment score of signaling pathways from the GSEAs that were performed on the phosphoproteomics data was examined. A negative correlation and a nonlinear relationship were discovered between the Notch reporter intensity values and the phosphopeptide intensities and the enrichment score of the Kyoto Encyclopedia of Genes and Genomes (KEGG) Notch signaling pathway, respectively (Fig. 5B). This further suggests that the discoveries from the phosphoproteomic analysis are reproducible by other methodologies.

**Fig. 6:**
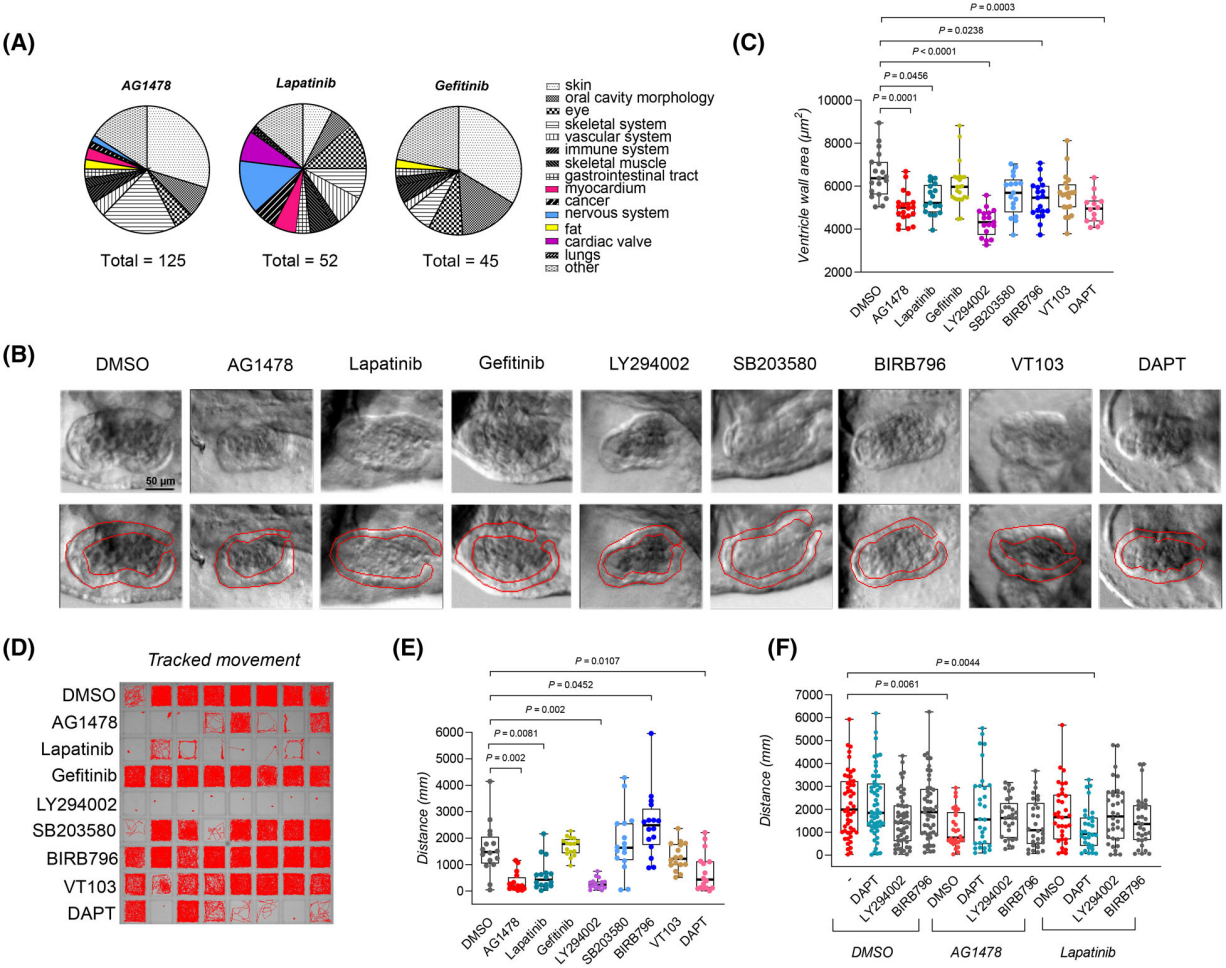
Phenotypic changes induced by inhibitors targeting the ErbB receptors and the signaling pathways differentially perturbed by the ErbB inhibitors. (A) Enriched phenotypes of ErbB inhibitor‐treated zebrafish embryos according to the GSEA on the global phosphoproteomics data. Only negatively enriched phenotypes were included and categorized into visualized subcategories. (B, C) Phase‐contrast images and quantification of the ventricle wall thickness of control or inhibitor‐treated zebrafish embryos at 4 dpf. Inhibitors targeting the ErbB receptors (AG1478 (10 μm, *n* = 20 embryos), lapatinib (30 μm, *n* = 17), gefitinib (30 μm, *n* = 23), the PI3K/Akt pathway (LY294002, 30 μm, *n* = 17), the p38 MAPK pathway (SB203580, 30 μm, *n* = 17 BIRB796, 30 μm, *n* = 19), the Hippo/YAP pathway (VT103, 30 μm, *n* = 20) and the Notch pathway (DAPT, 50 μm, *n* = 14)) were examined. Kruskal–Wallis ANOVA and Dunnett's multicomparison test were used for statistical testing. The red line indicates ventricle wall area. Scale bar, 50 μm. (D, E) Motility of control or inhibitor‐treated zebrafish embryos at 4 dpf. Inhibitors targeting the ErbB receptors (AG1478 (10 μm), lapatinib (30 μm), gefitinib (30 μm)), the PI3K/Akt pathway (LY294002, 30 μm), the p38 MAPK pathway (SB203580, 30 μm, BIRB796, 30 μm), the Hippo/YAP pathway (VT103, 30 μm) and the Notch pathway (DAPT, 50 μm) were analyzed. The tracked path (D) and the quantifications of the traveled distance are visualized (E). Kruskal–Wallis ANOVA and Dunnett's multicomparison test were used for statistical testing. Data pooled from two replicate experiments. (F) Motility of co‐treated zebrafish embryos at 4 dpf. Embryos treated with the inhibitors targeting the PI3K/Akt (LY294002, 1 μm), the p38 MAPK (BIRB796, 0.50 μm), Notch (DAPT, 0.20 μm) and ErbB (AG1478, 0.25 μm; lapatinib, 0.70 μm) pathways were examined alone or in combination in low doses. The quantifications of the traveled distance are visualized. Kruskal–Wallis ANOVA and Dunn's multicomparison test were used for statistical testing. Data pooled from three replicate experiments. One dot corresponds to one zebrafish embryo. In the boxplots, the line denotes the median, the box the interquartile range, and whiskers the whole range of values. Only *P*‐values of *P* < 0.10 are plotted in the graphs.

A GSEA of the ontologies of the human phenotype ontology database [28] was performed on the phosphoproteomic data (Fig. 6A, Tables S5 and S6). Differences in the phenotypes associated with the phosphorylation changes induced by the ErbB inhibitors were observed. As also previously reported [18, 19], myocardial and neurological phenotypes were only associated with AG1478 and lapatinib treatment. Since the phosphoproteomic changes induced by the treatment of these inhibitors were markedly different, we wanted to explore whether blocking the pathways differentially regulated by AG1478 and lapatinib would result in similar neurological and myocardial phenotypes in embryonic zebrafish to AG1478 or lapatinib treatment. To analyze the myocardial phenotype, zebrafish embryos were treated for 2 days with the ErbB inhibitors and inhibitors targeting the PI3K/Akt (LY294002), p38 MAPK (SB203580 and BIRB796), Notch (DAPT), and Hippo/YAP (VT103) pathways. The inhibitors were used at concentration ranges previously reported to be effective in inducing phenotypic differences in zebrafish embryos [29, 30, 31, 32]. The ventricle wall area was measured with *in vivo* imaging of zebrafish embryo hearts at 4 dpf (Fig. 6B). A distinct difference in the ventricle wall growth was detected by the treatment of inhibitors targeting the PI3K/Akt, p38 MAPK, and Notch signaling along with AG1478 and lapatinib treatment (Fig. 6C, Table S6). To analyze the differential effects on neurological phenotypes suggested by the GSEA (Fig. 6A), we exposed zebrafish embryos to the inhibitors and carried out motility assays (Fig. 6D) as these assays are widely used to assess diverse neurological phenotypes in zebrafish larvae and integrate many neurobiological functions at whole‐organism level [33]. The inhibitors targeting PI3K/Akt, p38 MAPK, and Notch signaling affected the motility of the zebrafish embryos along with AG1478 and lapatinib (Fig. 6E, Table S6). This, along with the results of the phosphoproteomic analysis, suggests that these differentially regulated pathways are involved in the phenotypic changes induced by AG1478 and lapatinib treatment.

To further explore this hypothesis, the effect of the co‐treatment of AG1478 or lapatinib with inhibitors targeting either the PI3K/Akt (LY294002), p38 MAPK (BIRB796), or Notch (DAPT) signaling pathway was analyzed. We hypothesized that the involvement of different pathways would affect sensitivity for different inhibitors. This hypothesis was tested by using low concentrations of inhibitors alone or as cotreatments. Lower concentrations of the inhibitors were used to reduce mortality and to facilitate the observation of potential synergistic effects. The cotreatment of lapatinib with DAPT reduced the motility of the embryos beyond that of single‐agent treatment, whereas this effect was not seen in cotreatment with AG1478 and DAPT (Fig. 6F, Table S6). Taken together, these results suggest that AG1478 and lapatinib induce similar phenotypes but perturb different signaling pathways. Inhibition of these pathways induces similar phenotypes to lapatinib or AG1478 treatment in zebrafish embryos.

## Discussion

We carried out in‐depth phosphoproteomic analysis of three structurally related ErbB inhibitors (AG1478, lapatinib, gefitinib) at the whole‐organism level on zebrafish embryos. Despite some differences to mammalian models, the zebrafish embryos are a versatile and efficient *in vivo* model for drug discovery and research for both efficacy and toxicological studies [15]. The effects of small molecule inhibitors on phosphoproteome have been rarely observed at a whole‐organism level although these compounds are known to affect the organism globally. More targeted phosphoproteomic analyses may not detect all perturbed pathways since the perturbation might be limited to unprobed organs or arise from the intercommunication between tissues. The analysis of whole organisms may, therefore, offer new insight into the biological activity of compounds despite its sensitivity limitations. The whole‐organism analysis and more targeted tissue or cell‐type‐specific analyses should be considered complementary approaches to comprehensively understand the biological properties of tested compounds. It is also good to note that this whole‐organism mass spectrometry analysis of phosphoproteome is not suitable for analysis of compound binding profiles *in vivo* but rather for downstream analyses of compounds.

The ErbB inhibitors we selected to investigate all share the same core structure, which targets the ATP binding pocket of the kinase domain of ErbB receptors [2]. Despite these similarities, their effects on the global phosphoproteome were significantly different. These changes may be due not only to differences in on‐target and off‐target profiles but also to absorption, distribution, metabolism, excretion, and drug dose. Regardless of the underlying root cause, these differences in effects on downstream signaling may also explain the observed differential biological effects [18, 19]. Our data, however, indicated that the human ErbB inhibition profile explained a significant proportion of the observed signaling changes in zebrafish embryos. The accurate inhibition profiles of these inhibitors toward zebrafish ErbB receptors remain to be elucidated. This may require the development of more sensitive targeted mass spectrometry assays. ErbB inhibitors differ in their efficacy and toxicity also in clinical settings [34], and one underlying cause could be differential effects on cell signaling. The global phosphoproteomic approach we employed here could be more widely used to analyze the differential signaling effects of closely related compounds. Especially the pathways for which no specific tools exist could be probed in this manner. This interpretation is supported by our experiments where we observed several differentially affected pathways that do not have these specific tools necessary available. With our approach, we were able to link the p38 MAPK, Notch, and PI3K/Akt‐signaling pathways into ErbB‐inhibitor‐induced neurological and myocardial phenotypes.

We further utilized the global phosphoproteomic analysis results by carrying out extensive analyses to identify altered pathways. Indeed, we were able to confirm several identified pathways using phospho‐specific antibodies. These antibodies exist only for a limited number of potential targets, and antibodies suited for work with zebrafish material are even more scarce [35]. Many of the phosphorylation site‐specific antibodies suitable for zebrafish material also detect the phosphorylation in several protein family members, further complicating the interpretation of the results. With the MS‐based phosphoproteomic analysis, we can more frequently detect the phosphorylation of the exact modified protein. In some rare cases, such as Notch, a reporter fish line may also be available. The clear validation of our findings from phosphoproteomic analysis with some phosphoresidue‐specific antibodies and the Notch‐reporter line, however, indicated that phosphoproteomic analysis of embryos is a sensitive and reliable tool to estimate the activity of various signaling pathways at the whole‐embryo level. Due to the lack of target‐specific tools, this observation is vital, since it indicates that the signaling activities of previously almost unprobeable pathways can be reliably measured with this approach.

As an intriguing finding, we observed the upregulation of the p38 MAPK and mTORC1‐mediated signaling pathways in zebrafish embryos by lapatinib but not by AG1478 and gefitinib. As is well‐documented, lapatinib has been shown to inhibit p38 MAPK phosphorylation in MDA‐MB‐231 breast cancer tumorspheres [17]. However, lapatinib has also been documented to activate the p38 MAPK in triple‐negative breast cancer cells by downregulating miR‐7 and increasing Raf‐1/ERK and IL‐6 expression [36]. This suggests that the lapatinib‐induced p38 MAPK activation can be context‐dependent and differentially regulated *in vivo* at the whole‐organism level. Similarly, lapatinib also induced mTORC1/S6K activation in zebrafish embryos (Fig. 2B), which could be explained by the downregulation of the well‐known negative feedback loop involving the S6K. In the negative feedback loop, the phosphorylation of the insulin receptor substrate 1 (IRS‐1) by S6K impairs the function of IRS‐1, which reduces the activation of PI3K and downstream components, such as Akt [37]. Additionally, the observed differential regulation of pathways could also be partially related to off‐target effects of the kinase inhibitors, as they often interact with multiple kinases in different contexts. Lapatinib, as a dual EGFR/ErbB2 inhibitor, has been reported to efficiently induce apoptosis by upregulating the proapoptotic TRAIL death receptors DR4 and DR5 independent of EGFR and ErbB2 [38]. Gefitinib has been shown to inhibit a long list of off‐target kinases including ErbB4, PIM1, MAPK10, and MAPK14 [39]. Similarly, AG1478, an EGFR/ErbB2/ErbB4 inhibitor, has been reported to modulate other kinases including the protein kinase CK2 [40]. These observations complicate the interpretation of our findings and off‐target effects should be considered while conclusions are drawn from phosphoproteomic whole‐organism analyses of compound actions.

We linked the observed global phosphoproteomic changes to phenotypes by performing phenotype enrichment analyses on the phosphoproteome profiles and cardiac and neurological phenotypic analyses with inhibitory compounds. From the enrichment analyses of the phosphoproteomic profile alone, we were able to predict the affected organs. Moreover, the predictions reflected previous observations of cardiac and neurological effects associated with lapatinib and AG1478 treatment, but not with gefitinib treatment [12, 19, 41, 42, 43, 44, 45]. Skin rash and diarrhea have been identified as the most commonly reported adverse effects of ErbB inhibitor treatments [45, 46]. The organ systems from which these symptoms originate (skin and the gastrointestinal tract) were indicated as affected by the enrichment analyses. The enrichment analyses, however, were unable to find an association between the observed phosphoproteome changes and the hepatotoxicity related to gefitinib or lapatinib treatment [46, 47]. This may be because the onset of ErbB inhibitor‐induced hepatotoxicity has been observed in humans only after repeated treatment or in combination with other therapeutic agents [47, 48]. In addition to the effects observed in the clinic, the phenotypic enrichment analyses also primarily reflected the known phenotypes of the embryonic knockout of the target ErbB receptors in mice [7, 8, 9, 10, 11, 12, 13, 14]. These observations indicate that the global phosphoproteomic profiling could be successfully used as an initial screen not just to identify key affected downstream pathways but also to identify affected organ systems of small molecule compound treatments. This strategy then could guide which organ systems should be subjected to more targeted analyses to identify effects on specific cell types. The methodology for such organ‐specific phosphoproteomic analysis is already well‐established in rodents [49].

Interestingly, our data also indicate that lapatinib and AG1478 had similar phenotypic effects, but they differentially affected signaling pathways in the phosphoproteomic analysis. Both lapatinib and AG1478 reduced the overall movement and ventricle wall growth of zebrafish embryos, but differentially perturbed the PI3K/Akt, p38 MAPK, and Notch pathways. Inhibition of these differentially regulated pathways had a similar phenotypic response to lapatinib and AG1478 treatment, suggesting that the differential regulation of these pathways is involved in the induced phenotypes. Interestingly, AG1478 and lapatinib‐treated embryos also had differential sensitivity to Notch inhibition by DAPT. The embryos were more sensitive to Notch inhibition after lapatinib treatment, and the sensitivity to Notch inhibition after lapatinib treatment has been additionally observed in the breast cancer context [50, 51]. This further indicates that Notch signaling plays a differential role in lapatinib and AG1478‐induced phenotypes. These observations are not, however, unexpected since lapatinib and AG1478 have different ErbB selectivity profiles. The ErbB selectivity profile, however, fails to explain the reason why the signaling changes induced by an EGFR/ErbB2 inhibitor were consistently the most unique in two separate inhibitor panels that, in addition, included a selective EGFR inhibitor and pan‐ErbB inhibitor. Furthermore, the ErbB2/ErbB4 heterodimer that is considered responsible for the cardiac and neuromuscular phenotypes is targeted both by lapatinib and AG1478, despite the low affinity of lapatinib with the ErbB4 kinase domain [7, 8, 12, 19]. It remains to be established whether the signaling changes we observed between lapatinib and AG1478‐treated embryos are the result of compensatory ErbB4 homodimer signaling or off‐target effects.

Our observations that an EGFR/ERBB2 inhibitor (lapatinib and AG825) and a pan‐ERBB inhibitor (AG1478, afatinib and neratinib) treatment induce differential global signaling changes may be of special interest since both an EGFR/ERBB2 inhibitor (lapatinib) and pan‐ERBB inhibitor (neratinib) are currently being used in the clinic as a therapy for HER2+ breast cancer [52]. The differential global signaling changes may contribute to the different toxicity profiles and efficacy of these treatments and perhaps could aid in designing co‐treatment strategies. Our observations of similar phenotypes but differential signaling activities overall may open interesting future avenues in analyses of compounds with overlapping primary targets.

## Materials and methods

### Zebrafish husbandry

Zebrafish were maintained in Aqua‐Schwarz (Göttingen, Germany) stand‐alone units with a 12 h : 12 h light : dark rhythm and fed with Skretting GEMMA micro dry food (Planktovie, Marseille, France). The water used in fish systems was municipal tap water filtered through an activated charcoal filter. After filtration, the water had the following properties: pH 7.2, KH (dKH) 3, GH (dGH) 4, CO_2_ 6, NH_4_ (mg·L^−1^, p.p.m.) < 0.05, NO_2_ (mg·L^−1^, p.p.m.) < 0.01, NO_3_ (mg·L^−1^, p.p.m.) 3, PO_4_ (mg·L^−1^, p.p.m.) 0.05, SiO_2_ (mg·L^−1^, p.p.m.) 1.6, Fe (mg·L^−1^, p.p.m.) < 0.02, Cu (mg·L^−1^, p.p.m.) < 0.05, O_2_ (mg·L^−1^, p.p.m.) 10, and conductivity 180 μS. The embryos were collected through natural spawning using 1.7 L Sloping Breeding Tanks (Tecniplast, Buggugiate, Italy). After collection, the embryos were maintained in E3 medium in a 28.5 °C incubator until used in experiments. The use of zebrafish embryos under 5 days of age is not considered an animal experiment, and hence does not require an ethical license according to Finnish and EU legislation. Adult zebrafish were housed under facility license MMM/465/712‐93 (Ministry of Agriculture and Forestry of Finland).

### Western analysis

Zebrafish embryos of AB line at 3 dpf were treated for 1 h with 10 μm of AG1478, 30 μm of Lapatinib, 30 μm of Gefitinib, 20 μm of Erlotinib, 20 μm of AG825, 20 μm of Afatinib, 20 μm of Neratinib, or DMSO. After the treatment, the embryos were mechanically dissociated with a motorized pestle in 100 μL of 2× Laemmli buffer. Five embryos were pooled per sample. The lysed samples were incubated for 5 min at 100 °C before 30 μL of the sample was loaded to SDS/PAGE gels. Tris‐Glycine‐based 10% acrylamide gels were cast and used. The samples were run on the gel in Tris‐Glycine running buffer for 1.5 h at 120 V. The separated proteins were consequently transferred to a nitrocellulose membrane immersed in methanol‐based Tris‐glycine transfer buffer on ice with 110 V for 1.5 h at 4 °C. The consequent steps before detection were all performed under agitation. The membranes were blocked with 5% skimmed milk in TBST (Tris‐buffered saline pH 7.4 with 0.1% Tween 20) for 30 min and incubated with primary antibodies for 18 h in 5% BSA in TBST at 4 °C. The membranes were washed four times for 5 min with TBST and incubated for 1 h with secondary antibodies that were diluted to 5% skimmed milk in TBST. The membranes were washed four times for 5 min with TBST, and the signal was detected. The antibodies were stripped from the membrane with a glycine‐based stripping buffer pH 2.2 with 2% SDS under agitation. The membranes were probed with anti‐phospho‐p38 MAPK T180/Y182 (#9211; Cell Signaling Technologies, Danvers, MA, USA), anti‐phospho‐p38 T180/Y182 (#4511; Cell Signaling Technologies), anti‐phospho‐p44/42 MAPK (Erk1/2) Thr202/Tyr204 (#9101; Cell Signaling Technologies), anti‐phospho‐Akt Ser473 D9E (#4060; Cell Signaling Technologies), anti‐p44/42 MAPK (Erk1/2) (9102; Cell Signaling Technologies), anti‐Akt (pan) 40D4 (#2920; Cell Signaling Technologies), and anti‐β‐actin (A5411; Sigma‐Aldrich, Merck KGaA, Darmstadt, Germany) primary antibodies at 1 : 1000 dilutions. Residue numbers here and in the figures refer to the numbering of human proteins. IR‐conjugated secondary antibodies at 1 : 15 000 dilutions and the Li‐Cor Odyssey system were used for signal detection. For total protein detection, MemCode Reversible Protein Stain Kit (#24580; Thermo Fisher Scientific, Waltham, MA, USA) was utilized according to the manufacturer's instructions.

The phosphoresidue‐specificity of antibodies was examined by homogenizing embryos in lysis buffer (0.1% Triton X‐100, 1 mm EDTA, 10 mm Tris–HCl, pH 7.4) supplemented with cOmplete, Mini, EDTA‐free Protease Inhibitor Cocktail (04693159001; Roche) or Halt™ Protease and Phosphatase Inhibitor Cocktail (78440; Thermo Scientific) The lysates were incubated at +37 °C for 90 min to activate endogenous phosphatases and subjected to western analysis. To examine the phosphoresidue‐specificity of antibodies with a recombinant phosphatase, zebrafish embryos were lysed in lysis buffer (0.1% Triton X‐100, 1 mm EDTA, 10 mm Tris–HCl, pH 7.4) supplemented with cOmplete, Mini, EDTA‐free Protease Inhibitor Cocktail (04693159001; Roche Diagnostics GmbH, Mannheim, Germany). The lysate was added to the reaction mixture of the FastAP kit (#EF0651; Thermo Fisher Scientific, Bremen, Germany), which either contained or did not contain the FastAP enzyme. The reaction mixture containing the FastAP enzyme was incubated at +37 °C for 60 min, and both reaction mixtures were supplemented with 6 × Laemmli buffer to obtain a 1 × Laemmli solution. The samples were denatured at 100 °C for 5 min and subjected to western analysis.

### Ventricle growth assay

Zebrafish embryos of casper line at 2 dpf were treated for 2 days with 10 μm of AG1478 (10010244; Cayman Chemicals, Ann Arbor, MI, USA), 30 μm of lapatinib (S2111; Selleckchem, Houston, TX, USA), 30 μm of gefitinib (13166; Cayman Chemicals), 30 μm of LY294002 (70920; Cayman Chemicals), 30 μm of SB203580 (559389; Sigma‐Aldrich), 30 μm of BIRB796 (HY‐10320; Axon Medchem, Groningen, the Netherlands), 30 μm of VT103 (HY‐134955; MedChemExpress EU, Sollentuna, Sweden), 50 μm of DAPT (2634; Bio‐Techne Ireland Ltd, Dublin, Ireland), or DMSO (final concentration 1%). The embryos were anesthetized with buffered tricaine (200 mg·L^−1^) and imaged live with a Zeiss AxioZoom stereomicroscope (Carl Zeiss AG, Oberkochen, Germany) using a Hamamatsu sCMOS Orca Flash4.0 LT + camera with 40 frames per second acquisition to capture different phases of the cardiac cycle. The ventricle wall area was estimated by multiplying the average ventricle thickness with the average ventricle perimeter. The ventricle perimeter was measured both in one diastole frame and one systole frame, and the average perimeter over these states was reported. The ventricle wall thickness was only measured in one systole frame. The ventricle thickness and perimeter were measured from the high‐speed videos with fiji [53].

### Motility assay

Zebrafish embryos of the AB line at 2 dpf were treated for 2 days with 10 μm of AG1478, 30 μm of lapatinib, 30 μm of gefitinib, 30 μm of LY294002, 30 μm of SB203580, 30 μm of BIRB796, 30 μm of VT103, 50 μm of DAPT, or DMSO (final concentration 1%). For the cotreatment analyses, the embryos at 2 dpf were treated for 2 days with 0.25 μm of AG1478, 0.70 μm of lapatinib, 1 μm LY294002, 0.50 μm BIRB796, 0.20 μm DAPT, or DMSO alone or in the indicated combinations. The motility of the embryos was measured at 28.5 °C with the Daniovision instrument (Noldus IT, Wagenigen, the Netherlands) equipped with a Temperature Control Unit. Tracking and analysis were performed with integrated ethovision xt software (Noldus IT, Wagenigen, the Netherlands). Outliers were removed with the method of ROUT with *Q* = 10%.

### Notch‐reporter assay

Zebrafish embryos of the tp1:Venus‐pest reporter line [24] were treated for 1 day with 10 μm of AG1478, 30 μm of lapatinib, 30 μm of gefitinib, 20 μm of Erlotinib, 20 μm of AG825, 20 μm of Afatinib, 20 μm of Neratinib, or DMSO at 2 dpf. The treated embryos were transferred into a 96‐well plate and imaged with a Nikon Eclipse Ti‐2 with 475/28 nm LED excitation, Quad filter (84000v2; Chroma Technology, Olching, Germany) and a 2× Nikon Plan‐Apochromat (NA 0.06) objective. The intensity of the fluorescent signal was measured from the images with fiji [53]. The lens was not included in the intensity measurements in the eye, and the intensity of the area outside the embryo was measured as the background. The intensity values were normalized against the background signal, and the background signal value was reduced from the final normalized intensity values.

### Phosphopeptide enrichment

Zebrafish embryos of the casper line at 3 dpf were treated for 1 h with 10 μm of AG1478, 30 μm of lapatinib, 30 μm of gefitinib, or DMSO. After treatment, the embryos were mechanically dissociated with a motorized pestle in 8 m urea in 50 mm Tris–HCl, pH 8. Twenty embryos were pooled per sample. The proteins in the sample were reduced with 10 mm d,l‐dithiothreitol in 50 mm Tris–HCl, pH 8, and alkylated with 40 mm iodoacetamide in 50 mm Tris–HCl, pH 8. Samples were digested overnight with sequencing‐grade modified trypsin (Promega, Madison, WI, USA). Peptides were desalted with a Sep‐Pak C18 well plate (Waters, Milford, MA, USA). The phosphopeptide enrichment was performed with a High‐Select TiO_2_ Phosphopeptide Enrichment for complex samples kit (Thermo Fisher Scientific) according to the manufacturer's protocol. The phosphopeptide enrichment was performed with 500 ng of protein per sample.

### Mass spectrometry

The MS/MS analysis was performed in the Proteomics Core of Turku Bioscience Centre, partially supported by Biocenter Finland. The enriched phosphopeptides were resuspended into 0.1% formic acid before injection to liquid chromatography electrospray ionization tandem mass spectrometry (LC‐ESI‐MS/MS) protein analysis. The LC‐ESI‐MS/MS analyses were performed on a nanoflow HPLC system (Easy‐nLC1000; Thermo Fisher Scientific) coupled to the Q Exactive HF mass spectrometer (Thermo Fisher Scientific) equipped with a nano‐electrospray ionization source. Peptides were first loaded on a trapping column and subsequently separated inline on a 15 cm C18 column (75 μm × 15 cm, ReproSil‐Pur R13aq 3 μm 120 Å C18‐AQ; Dr. Maisch HPLC GmbH, Ammerbuch‐Entringen, Germany). The mobile phase consisted of water with 0.1% formic acid (solvent A) and acetonitrile/water (80 : 20 (v/v)) with 0.1% formic acid (solvent B). A 90 min 2‐step gradient from 7% to 23% in 68 min and to 36% of eluent B in 22 min, followed by a wash stage with 100% of eluent B, was used to elute peptides. MS data was acquired automatically by using Thermo xcalibur 4.1 software (Thermo Fisher Scientific). In the DIA method, a duty cycle contained one full scan (400–1000 *m/z*) and 40 DIA MS/MS scans covering the mass range 400–1000 with isolation width 15 *m/z*.

Additionally, six gas phase fractionation (GPF)–DIA acquisitions [54] were acquired of a phosphopeptide sample pool (60 000 precursor resolution, 30 000 fragment resolution, AGC target of 1e6, max IT of 50 ms, NCE of 30) using 3 *m/z* precursor isolation windows with optimized window placements for phosphopeptides by EncyclopeDIA (i.e., 400.362–520.416 *m/z*, 500.407–620.462 *m/z*, 600.453–720.507 *m/z*, 700.498–820.553 *m/z*, 800.544–920.598 *m/z*, and 900.589–1020.644 *m/z*) [55].

### Mass spectrometry data analysis

Data processing was carried out by the spectronaut 14.7. software (Biognosys AG, Schlieren, Switzerland). A spectrum library was generated from analyzed samples and GPF‐DIA acquisitions using the Pulsar search engine in Spectronaut with a FASTA file of *Danio rerio*, downloaded from SwissProt (23.11.2020). The following modifications were used in the Spectronaut analysis: Carbamidomethyl C, Acetyl Protein N‐term, Oxidation M, and Phospho STY. The library was used to perform DIA library‐based analysis in Spectronaut to search sample raw files. The LFQ intensities of the phosphopeptides were sample‐wise normalized to the sum of the LFQ intensities of all peptides, and these normalized intensities were used for statistical testing. Mackskill's test was used for differential expression analysis with the different replicate experiments assigned as covariates. The *P*‐values were FDR‐corrected. Phosphopeptides with the *Q*‐value below 0.05 and fold change above 1.5 or below 0.66 against the DMSO control treatment were considered significantly differential abundances.

### Bioinformatic analyses

The overrepresentation analyses were performed with the Panther Overrepresentation Test (Release 2023‐10‐17) [56] or gProfiler (database updated on 2024‐01‐25) [57]. Reactome version 85 (Release 2023‐05‐25) [58], KEGG (Release 2024‐01‐22) [59], and WikiPathways (Release 2024‐01‐01) [60] databases were sourced for pathway annotations. The gene set enrichment analyses were performed with GSEA v 4.3.2 [23] with classic analysis from ranked *Q*‐value weighted fold‐change values against the DMSO control. The c2.all.v2023.2 and c5.hpo.v2023.2 gene sets from MsigDB v2023.2.Hs [61] were used for the GSEA. The human phosphoproteomics datasets were acquired from the publications of Koch *et al*., Wang *et al*., Imami *et al*., Sidhanth *et al*., Herman *et al*., and Klaeger *et al*. [62, 63, 64, 65, 66, 67]. The overrepresented pathways with FDR below or equal to 0.10 were used to analyze the pathway overlap.

### Statistical analyses

For statistical testing, graphpad prism v10.1 (GraphPad Software, Boston, MA, USA) and R v4.3.1 [68] were utilized. The equality of variance and normality assumptions were tested with Bartlett's, Brown–Forsythe, Kolmogorov–Smirnov, Shapiro–Wilk, D'Agostino–Pearson, and Pearson–Omnibus tests, and parametric or nonparametric testing was used accordingly. For parametric testing, one‐way ANOVA, Welch's ANOVA, and two‐tailed *t*‐test were utilized. For nonparametric testing, Kruskal–Wallis ANOVA and Wilcoxon rank‐sum tests were utilized. For ANOVA *post hoc* analyses, either Dunn's or Dunnet's multicomparison tests were utilized. The correlation analyses were performed with one‐tailed Pearson's correlation analysis. The nonlinear relationship was estimated by fitting a quadratic curve with least squares estimation. The *P*‐value for the *R* ^2^ of the quadratic curve was calculated with an *F*‐test using r v4.3.1 [68]. The χ^2^ test was used to estimate the statistical significance of dataset overlap.

### Data visualization

graphpad prism v10.1 was primarily used for data visualization. The heatmap of differentially regulated phosphopeptides was generated with matlab 2022b (MathWorks, Natick, MA, USA). Hierarchical clustering and data standardization were used for the heatmap visualization. The heatmaps for phosphoprotein and pathway overlaps were illustrated with morpheus (Broad Institute, https://software.broadinstitute.org/morpheus/). The illustrations were produced with biorender.com.

## Conflict of interest

The authors declare no conflict of interest.

## Author contributions

KV, JÖ, IP, and KE contributed to conceptualization. KV, JM, and IP contributed to methodology. KV, NV, IP, and AJ contributed to validation. IP, KV, and JM contributed to formal analysis. KV, IP, NV, JÖ, AJ, PH, IF, and ZS contributed to investigation. KV and JM contributed to data curation. KV and IP contributed to writing—original draft. KV, IP, JÖ, NV, CS, KE, AJ, JM, ZS, and IF contributed to writing—review and editing. KV and IF contributed to visualization. IP, CS and KE contributed to supervision. IP contributed to project administration. IP, KE, CS and KV contributed to funding acquisition.

## Supporting information


**Table S1.** A table of detected unique phosphopeptides from the embryos with protein mass spectrometry using data‐independent acquisition with tandem mass spectrometry (MS/MS).
**Table S2.** A table of quantified phosphopeptides that were significantly altered between treatments.
**Table S3.** Gene set enrichment analyses of the dysregulated signaling pathways.
**Table S4.** Sequence similarity of human and zebrafish ERBB kinase domains.
**Table S5.** A gene set enrichment analysis of the ontologies of the human phenotype ontology database of the phosphoproteomic data.
**Table S6.** Numerical source data for graphs 2G, 2H, 3A, 3B, 4A, 4B, 5A, 6A, 6C, 6E, and 6F.

## Data Availability

Mass spectrometric data are available in ProteomeXchange Consortium via the PRIDE [69] partner repository with the accession code PXD050396. Source data for figures and sequence alignments are available in Tables S1–S6. Other source data is available from authors upon reasonable request.
