# Supplementary material for: Phosphoproteomic analysis reveals the diversity of signaling behind ErbB‐inhibitor‐induced phenotypes
Source: FEBS J. 2025 Jul 24;292(24):6557–75. doi: 10.1111/febs.70197 (PMC12713336; doi:10.1111/febs.70197)
Supplement: Supplementary file 1 — Table S1. A table of detected unique phosphopeptides from the embryos with protein mass spectrometry using data‐independent acquisition with tandem mass spectrometry (MS/MS). Table S2. A table of quantified phosphopeptides that were significantly altered between treatments. Table S3. Gene set enrichment analyses of the dysregulated signaling pathways. Table S4. Sequence similarity of human and zebrafish ERBB kinase domains. Table S5. A gene set enrichment analysis of the ontologies of the human phenotype ontology database of the phosphoproteomic data. Table S6. Numerical source data for graphs 2G, 2H, 3A, 3B, 4A, 4B, 5A, 6A, 6C, 6E, and 6F. [file FEBS-292-6557-s001.zip › TablesS1-S6_Legends.pdf]

Supporting Information –Legends of Supplemental Tables

Supplemental Table 1. *A table of detected unique phosphopeptides from the embryos with protein mass spectrometry using data-independent acquisition with tandem mass spectrometry (MS/MS).* The TiO_2 -enriched phosphopeptides were analyzed using liquid chromatography electrospray ionization tandem mass spectrometry (LC-ESI-MS/MS). Data analysis was carried out using Spectronaut software.

Supplemental Table 2. *A table of quantified phosphopeptides that were significantly altered between treatments.* Mackskill's test was used for differential expression analysis with the different replicate experiments assigned as covariates. The P-values were FDR-corrected.

Supplemental Table 3. *Gene set enrichment analyses (GSEAs) of the dysregulated signaling pathways.* Gene set enrichment analyses were performed with GSEA v 4.3.2 with classic analysis from ranked Q-value weighted fold-change values against the DMSO control.to identify the dysregulated signaling pathways from the whole dataset.

Supplemental Table 4. *Sequence similarity of human and zebrafish ERBB kinase domains.* A multiple sequence alignment on the kinase domains of human and zebrafish ErbB receptors was performed. The protein sequences of human and zebrafish ERBB kinase domains were acquired from Uniprot. The sequence identity was analyzed by multiple sequence alignment with Clustal Omega.

Supplemental Table 5. *A GSEA of the ontologies of the human phenotype ontology database of the phosphoproteomic data*. Gene-set enrichment analysis (GSEA) of the global phosphoproteomics data was carried out to predict affected phenotypes of ErbB inhibitor-treated zebrafish embryos. Only negatively enriched GSEA phenotypes were included.

Supplemental Table 6. *Numerical source data for graphs 2G, 2H, 3A, 3B, 4A, 4B, 5A, 6A, 6C, 6E and 6F.* The source data values used to make graphs. Data used in graphs in figures 2G, 2H, 3A, 3B, 4A, 5A, 6A, 6C, 6E and 6F are displayed in separate sheets. Each sheet is labeled with the figure number.
